# Chlamydia-induced reactive arthritis diagnosed during gout flares: A case report and cumulative effect of inflammatory cytokines on chronic arthritis

**DOI:** 10.1097/MD.0000000000017233

**Published:** 2019-10-04

**Authors:** Remi Sumiyoshi, Tomohiro Koga, Sosuke Tsuji, Yushiro Endo, Ayuko Takatani, Toshimasa Shimizu, Takashi Igawa, Masataka Umeda, Shoichi Fukui, Ayako Nishino, Shin-ya Kawashiri, Naoki Iwamoto, Kunihiro Ichinose, Mami Tamai, Hideki Nakamura, Tomoki Origuchi, Atsushi Kawakami

**Affiliations:** a Division of Advanced Preventive Medical Sciences, Department of Immunology and Rheumatology; b Center for Bioinformatics and Molecular Medicine, Nagasaki University Graduate School of Biomedical Sciences; c Medical Education Development Center, Nagasaki University Hospital; d Center for Comprehensive Community Care Education; e Department of Community Medicine, Unit of Advanced Preventive Medical Sciences, Nagasaki University Graduate School of Biomedical Sciences, Nagasaki, Japan.

**Keywords:** Chlamydia-induced reactive arthritis, cytokine profiles, gout, HLA-B27

## Abstract

**Rationale:**

The pathology of gouty arthritis and reactive arthritis (ReA) partially overlaps, and both diseases are characterized by the production of inflammatory cytokines associated with the activation of monocytes and macrophages. However, the precise cytokine profile of cases with a coexistence of both diseases is unknown, and there are few reports on the course of treatment in patients with both gouty arthritis and ReA.

**Patient concerns:**

A 39-year-old man with a recurrent episode of gouty arthritis presented prednisolone-resistant polyarthritis with high level of C-reactive protein (CRP). He had the features of gouty arthritis such as active synovitis of the first manifestation of metatarsophalangeal (MTP) joints and the presence of monosodium urate (MSU) crystals from synovial fluid. But he also had the features of ReA such as the presence of tenosynovitis in the upper limb, the positivity of human leukocyte antigen (HLA)-B27, a history of sexual contact and positive findings of anti-*Chlamydia trachomatis*-specific IgA and IgG serum antibodies.

**Diagnoses:**

He was diagnosed with HLA-B27 associated Chlamydia-induced ReA accompanied by gout flares.

**Interventions:**

He was treated with 180 mg/day of loxoprofen, 1 mg/day of colchicine, and 10 mg/day of prednisolone for gout flares. However, his polyarthritis worsened with an increased level of CRP (23.16 mg/dL). Accordingly, we added 500 mg/day of salazosulfapyridine followed by adalimumab (ADA) 40 mg once every 2 weeks.

**Outcomes:**

After starting ADA, the patient's symptoms and laboratory findings showed rapid improvement and he achieved clinical remission 1 month after initiation of ADA treatment. As of this writing, the patient's clinical remission has been maintained for >1 year.

**Lessons:**

This case suggests that with exacerbation of arthritis during gouty arthritis, coexistence with other pathologies such as peripheral spondyloarthritis should be considered, and early intensive treatment including tumor necrosis factor inhibitors may be necessary.

## 1 Introduction

Gouty arthritis is characterized by the manifestation of metatarsophalangeal (MTP) arthritis, which develops in response to monosodium urate (MSU) crystals.^[1]^ It sometimes occurs in large joints and multiple joints, and the prolonged inflammation that follows recurrent attacks of gout results in chronic gout.^[2]^ Clinical manifestations of chronic gout are characterized by chronic synovitis, bony erosions, cartilage damage, and tophi formation. Since these manifestations are like those of rheumatoid arthritis and spondyloarthritis (SpA), it is important to distinguish the symptoms from these diseases.

Reactive arthritis (ReA) is an acute arthritis that usually occurs following gastrointestinal, reproductive, or urinary tract infections, and it may exhibit extra-articular symptoms such as uveitis.^[3]^ It has been recognized as a form of seronegative SpA, and it presents with axial and/or peripheral musculoskeletal symptoms including enthesitis, dactylitis, back pain, and extra-articular signs. In patients with ReA, the presence of human leukocyte antigen (HLA)-B27 has been associated with a worse prognosis such as chronic SpA with radiographic changes.^[4,5]^

It is suggested that the pathology of gouty arthritis and ReA partially overlaps, and both diseases are characterized by the production of inflammatory cytokines associated with the activation of monocytes and macrophages. However, the precise cytokine profile of cases with a coexistence of both diseases is unknown, and there are few reports on the course of treatment in patients with both gouty arthritis and ReA. We herein report a case of Chlamydia-induced ReA diagnosed during gout flares that was successfully treated with a tumor necrosis factor (TNF) inhibitor.

## 2 Case report

A 39-year-old man with a family history of gout was diagnosed with gouty arthritis based on recurring arthritis in the first MTP joint of the left foot for 9 years. He was treated with non-steroidal anti-inflammatory drugs (NSAIDs) during episodes, but he was not treated for hyperuricemia. He took 45 g of alcohol every day and had overconsumption of purine-rich foods. In May 2017, he presented with arthralgia in the first MTP joints of both feet and in the left ankle. Despite treatment with NSAIDs, arthralgia expanded to the right knee and the right ankle in July 2017. He was referred to our department in August 2017 for further evaluation of polyarthralgia with a high level of C-reactive protein (CRP) (6.85 mg/dL).

On physical examination, His height was 175.5 cm and his weight was 67.4 kg. Body temperature was 36.5°C, blood pressure was 113/55 mm Hg, and pulse was 65 beats/min. There were no abnormal findings in the head and neck, chest, and abdomen. Tenderness was observed in the right shoulder joint, with swelling and tenderness in the right knee joint, left foot joint, and first MTP joints of both feet. There was no finding of skin rash or gouty tophus. Laboratory testing showed white blood cells (WBCs) 5900/μL, hemoglobin (Hb) 12.1 g/dL, platelets 35.6 × 10^4^/μL, CRP 4.85 mg/dL, erythrocyte sedimentation 71 mm/h, blood urea nitrogen 9 mg/dL, creatinine 0.81 mg/dL, uric acid 7.1 mg/dL, total protein 7.5 g/dL, albumin 3.8 g/dL, total bilirubin 0.9 mg/dL, aspartate aminotransferase 18 U/L, alanine aminotransferase 15 U/L, alkaline phosphatase 323 U/L, lactate dehydrogenase 128 U/L, creatine kinase 56 U/L, glucose 92 mg/dL, HbA1c 5.4%, rheumatoid factor 5.2 IU/mL, immunoglobulin (IgG) 1406 mg/dL, anti-Cyclic Citrullinated Peptide antibody 0.5 U/mL, anti-nuclear antibody <20 times, matrix metalloproteinase-3 286.7 ng/mL, anti-Ro/SS-A antibodies 405 μg/mL, tuberculosis (T-SPOT.TB) negative, and HLA-B7 and B27 positive. In addition, the examination of synovial fluid from the right knee joint showed the following results: WBC 4600/μL, and bacterial culture negative. MSU crystals were observed with polarized light microscopy.

**Figure 1:**
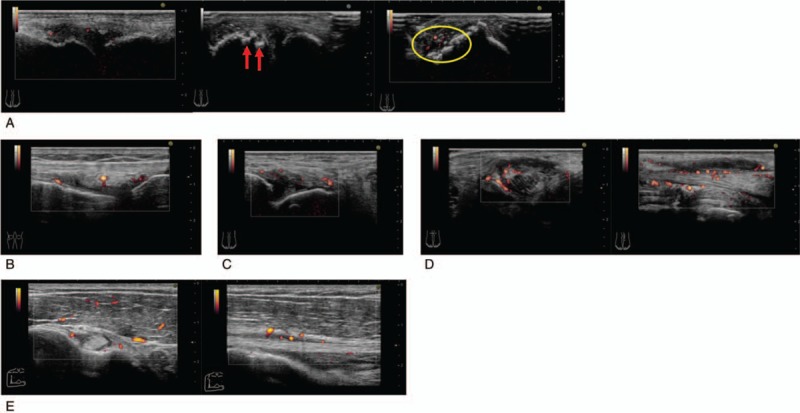
Musculoskeletal ultrasound (MSUS) at the first visit revealed active synovitis. (A) First metatarsophalangeal (MTP) joints of both feet had bone erosion (red arrows), crystal aggregates (yellow circle), and synovial thickening with power Doppler signals. (B) Right knee joint and (C) left ankle joint also had synovial thickening with power Doppler signals. (D) Left posterior tibial tendon and (E) right bicep tendon showed tendon sheath thickening with power Doppler signals.

Radiographic examination of hands, knees, and feet showed no bone erosion or joint space narrowing. A musculoskeletal ultrasound (MSUS) revealed synovitis with power Doppler signals and crystal aggregates in the first MTP joints of both feet, active synovitis in the right knee joint and left ankle joint, and tenosynovitis in the left posterior tibial tendon and right bicep tendon (Fig. 1). Magnetic resonance imaging of the sacroiliac joint showed no abnormality.

**Figure 2:**
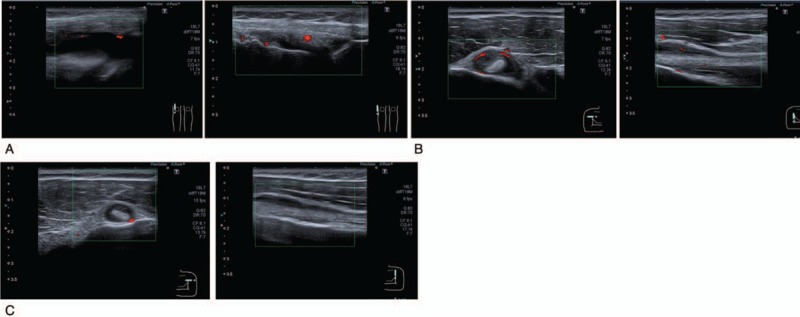
Musculoskeletal ultrasound (MSUS) findings after the initial treatment. MSUS findings when arthritis exacerbated with an increased level of C-reactive protein (CRP) (23.16 mg/dL), at September 2017. (A) Synovitis in the right knee joint and (B) tenosynovitis in the right bicep tendon exacerbated. (C) Tendon sheath thickening of the left biceps tendon newly appeared.

On the basis of these findings, the patient was diagnosed with chronic gouty arthritis, and 180 mg/day of loxoprofen and 1 mg/day of colchicine were introduced. However, 2 weeks later, the patient experienced polyarthritis with an increased level of CRP (23.16 mg/dL). MSUS findings revealed tenosynovitis in the bilateral bicep tendons and active synovitis in the right knee joint (Fig. 2A–C). Prednisolone 10 mg/day was added, but it was ineffective. After taking a detailed medical history, the patient reported infection opportunities of sexually transmitted diseases in March 2017. Serum anti-*Chlamydia trachomatis* IgA and IgG antibodies measured in September 2017 were all positive. In conjunction with HLA–B27 positivity, we considered that this patient developed Chlamydia-induced ReA during the course of gouty arthritis.

**Figure 3:**
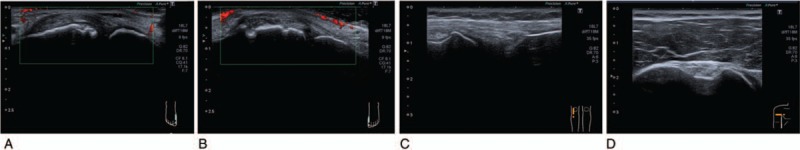
Musculoskeletal ultrasound (MSUS) findings 1 year after starting ADA, in November 2018. There was no active synovitis in the (A) right and (B) left first metatarsophalangeal (MTP) joint and (C) right knee joint. (D) There was no active tenosynovitis in the right bicep tendon.

**Figure 4:**
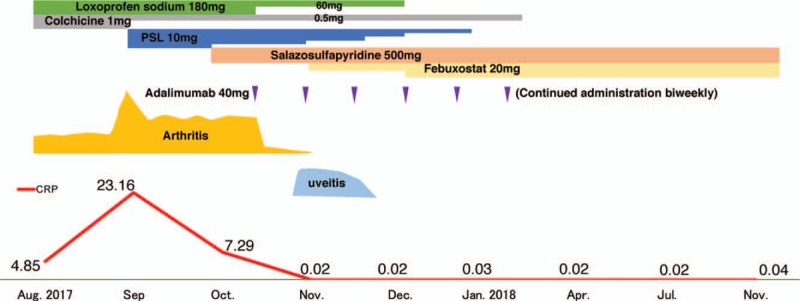
Clinical course of the patient. Graphs display the severity of arthritis, uveitis, and CRP as well as the treatment interventions. CRP = C-reactive protein, PSL = prednisolone.

Accordingly, 500 mg/day of salazosulfapyridine was added, and subsequently adalimumab (ADA) 40 mg once every 2 weeks was prescribed. After that treatment regimen, the patient's symptoms and laboratory findings showed rapid improvement. At 3 months after starting ADA, MSUS showed improvement of synovitis in most of joints but not in the left first MTP joint. We confirmed that his active synovitis and tenosynovitis was totally improved on the basis of MSUS findings at 6, and 12 months after starting ADA (Fig. 3A–D). During the clinical course, he developed mild unilateral anterior uveitis, but it improved with eye drop instillation. Also, febuxostat 10 mg/day was initiated for gouty arthritis, and loxoprofen, prednisolone, and colchicine were gradually tapered after the improvement of polyarthritis. The clinical course is shown in Figure 4. As of this writing, the patient's clinical remission has been maintained for >1 year.

We investigated the effect of TNF-α inhibition on the pathogenesis of the patient by analyzing his serum cytokines before and after the initiation of ADA. His levels of granulocyte colony-stimulating factor (G-CSF), interleukin (IL)-6, IL-8, CXCL1, and IL-18 were decreased after treatment, but the TNF-α level did not change after treatment (Table 1). In addition, the levels of serum TNF-α, G-CSF, CXCL1, and IL-6 were higher in the present case at diagnosis than in healthy controls (Table 1).^[6]^

**Table 1:**
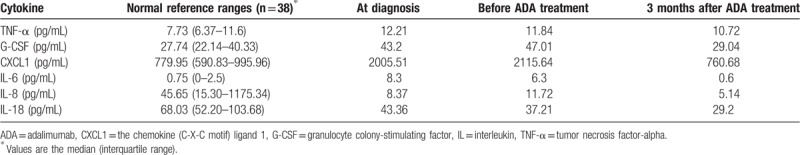
Changes in cytokines.

## 3 Discussion

Gouty arthritis sometimes co-occurs with sacroiliac arthritis,^[7]^ and it is important to distinguish it from SpA, including ReA. Prolonged hyperuricemia with poor control in this case may have contributed to the development of polyarthritis, because the duration of gout and degree of hyperuricemia were correlated with chronic gouty arthritis.^[8,9]^ However, the clinical course was not typical for chronic gouty arthritis in terms of the response to glucocorticoid and the distribution of his arthritis. Some findings were suggestive of gouty arthritis, such as hyperechoic small aggregates and synovial thickening with power Doppler signals of the first MTP joints by MSUS and MSU crystals detection from synovial fluid of his right knee joint. We considered that the coexistence of ReA associated with *Chlamydia* infection substantially contributed to his chronic polyarthritis. Our diagnosis was based on

1. the presence of tenosynovitis in the upper limb including the bilateral biceps tendons,
2. the positivity of HLA-B27,
3. a history of sexual contact and positive findings of anti-*C trachomatis*-specific IgA and IgG serum antibodies,
4. complication of unilateral anterior uveitis during the course, and
5. absence of palpable tophaceous deposits.

This patient did not show the positivity of PCR assay of urine sample for *C trachomatis*, because 6 months had passed since the infection opportunity.

Our patient was first treated with conventional synthetic disease-modifying anti-rheumatic drug for ReA, but a TNF inhibitor was introduced earlier than usual with explaining to the patient adequately because of the presence of HLA-B27, which has been reported as a poor prognostic factor for ReA.^[10]^ A previous report demonstrated that among the 10 patients with early onset ReA, TNF inhibitors were effective in nine cases, and partially effective in the one remaining case.^[11]^ Our patient's subjective symptoms showed rapid improvement, and he achieved clinical remission 1 month after initiation of ADA treatment. Consistent with clinical remission, the cytokine profile has also improved after treatment. The remission confirmed by MSUS has been maintained for over 1 year. However, one report showed that three out of six patients who achieved remission had recurrence after the discontinuation of TNF inhibitors^[11]^; thus, we will be cautious about discontinuing the TNF inhibitor in the present case.

Both gouty arthritis and ReA are types of arthritis in which autoantibodies are not detected, and some of the pathological conditions are shared. An activation of inflammasome plays an important role in gouty arthritis. During episodes of gout, monocytes and synoviocytes activate the nucleotide oligomerization domain-like receptor protein 3 inflammasome in response to MSU crystals.^[12]^ This activation leads to the release of active forms of IL-1β and IL-18. These cytokines activate nuclear factor κB signaling pathways via toll-like receptors that lead to increased amounts of IL-6, IL-8, and TNF-α.^[13]^

In ReA, HLA-B27 expressing monocytes and macrophages forms an abnormal homodimer by an infection. CD4-positive T cells recognize this abnormal homodimer and produce IFN-γ and IL-17.^[14]^ These cytokines activate monocytes, macrophages, osteoblasts, synovial fibroblasts, and vascular endothelium to release inflammatory cytokines such as IL-6 and TNF-α.^[15]^ Our patient had been suffering from gouty arthritis with hyperuricemia for years, and he subsequently had *Chlamydia* infection that triggered ReA. This suggested that the cumulative effects of the above two pathologies may have contributed to exacerbation of his arthritis.

In conclusion, this report presents a patient who developed *Chlamydia*-induced ReA during recurrent gout flares. This case suggests that when arthritis is exacerbated during gouty arthritis, physicians should consider a coexistence with other pathologies such as peripheral SpA. An early intensive treatment including TNF inhibitors may be necessary to control cumulative inflammatory cytokines resulting from the complication of gout and other arthritis.

## Author contributions

**Investigation:** Remi Sumiyoshi, Sosuke Tsuji, Yushiro Endo, Ayuko Takatani, Toshimasa Shimizu, Takashi Igawa, Masataka Umeda, Shoichi Fukui, Ayako Nishino, Shin-ya Kawashiri, Naoki Iwamoto, Kunihiro Ichinose, Mami Tamai, Hideki Nakamura, Tomoki Origuchi, Atsushi Kawakami.

**Supervision:** Tomohiro Koga.

**Writing – original draft:** Remi Sumiyoshi, Tomohiro Koga.

**Writing – review & editing:** Remi Sumiyoshi, Tomohiro Koga.
